# Collegiate badminton expertise is associated with interference-control and response-inhibition task performance: a cross-sectional expert–novice study

**DOI:** 10.3389/fpsyg.2026.1919654

**Published:** 2026-08-20

**Authors:** Yihan Zhang, Yuqing Wang, Xianghe Wang, Yixuan Peng, Zifu Shi, Wenzi Wang, Changchang Huang

**Affiliations:** 1 College of Physical Education, Hunan Normal University, Changsha, China; 2 Cognition and Human Behavior Key Laboratory of Hunan Province, School of Educational Science, Hunan Normal University, Yuelu, Changsha, China; 3 School of Psychology, Shanghai University of Sport, Shanghai, China; 4 Hunan SANY Polytechnic College, Changsha, Hunan, China

**Keywords:** collegiate badminton athletes, go/nogo tasks, inhibitory control, interference control, response inhibition, stroop task

## Abstract

**Introduction:**

Badminton places repeated demands on selecting task-relevant cues while withholding prepared actions, yet evidence for domain-general inhibitory-control differences associated with sport expertise remains mixed. This study examined whether collegiate badminton expertise was associated with performance on two non-sport-specific tasks assessing interference control and response inhibition.

**Methods:**

Sixty-eight right-handed university students (33 badminton experts and 35 novices) completed a color–word Stroop task followed by a Go/NoGo task. The primary Stroop outcome was the participant-level interference cost, calculated as incongruent minus congruent reaction time. Go/NoGo performance was characterized by hit rate, false-alarm rate, and a trial-level logistic mixed-effects model of NoGo errors. Corrected d' was treated as a secondary measure because of ceiling and floor constraints.

**Results:**

Experts showed a smaller Stroop interference cost than novices after reaction-time cleaning (13.6 vs. 44.6 ms; novice–expert difference = 31.0 ms, 95% CI [25.2, 37.3], *p* < 0.001, *d* = 2.33), with consistent conclusions from untrimmed, speed-adjusted, and inverse-efficiency analyses. Overall Stroop accuracy did not differ between groups. In the Go/NoGo task, experts responded faster on Go trials (259 vs. 290 ms), showed higher hit rates (0.9976 vs. 0.9825), and lower false-alarm rates (0.0049 vs. 0.0264). Trial-level modeling indicated lower odds of a NoGo error among experts (OR = 0.19, 95% CI [0.10, 0.36], *p* < 0.001). Corrected d' was also higher among experts; however, it was upper-censored, with 10 of 33 experts attaining the maximum value, limiting its interpretation at the individual level.

**Discussion:**

Collegiate badminton expertise was associated with better performance on both interference-control and response-inhibition tasks, suggesting that expertise-related differences may extend beyond badminton-specific perceptual and motor contexts. However, these associations should be interpreted as task- and sample-specific rather than as evidence of a unitary inhibitory-control advantage. Because the study was cross-sectional and lacked both an active-athlete control group and a pure processing-speed baseline, the findings do not establish whether the observed differences resulted from badminton training or pre-existing individual characteristics.

## 1 Introduction

As an open-skill sport, badminton is characterized by high shuttle speed, abrupt changes in landing position, rapid offensive-defensive transitions, and uncertainty about an opponent's next action. During rallies, players must use advance cues from body posture, racket-face orientation, striking rhythm, and early shuttle trajectory to anticipate the likely shot, while still remaining ready to revise a prepared response when a deceptive action or late trajectory change occurs. Meta-analytic evidence indicates that expert athletes show perceptual-cognitive advantages, particularly in cue use, anticipation, and decision making under sport-relevant constraints (Mann et al., 2007). Badminton-specific studies likewise link expertise to action inhibition, re-engagement processing, visuospatial attention, and conflict-task performance, although these findings remain task- and context-dependent (Chen et al., 2019; Chang et al., 2024; Hu, 2026). These features provide a theoretical rationale for examining task-based associations between badminton expertise and inhibitory-control performance, but they do not by themselves demonstrate transfer from training to domain-general cognition.

Inhibitory control is an important component of executive function and generally refers to the ability to suppress irrelevant information, prepotent responses, or impulsive behaviors in order to achieve current goals (Diamond, 2013). Research on executive function increasingly emphasizes the fractionation of control processes and the issue of task purity. A single laboratory task rarely measures one process in isolation, because task performance can also reflect processing speed, response mapping, alertness, task familiarity, strategy, and speed-accuracy tradeoffs. This point is particularly important in sport cognition, where an athlete's advantage on one task does not necessarily imply a global executive-function advantage. The classic unity-and-diversity framework proposes that executive-function tasks share common variance while also involving distinguishable components (Miyake et al., 2000). Friedman and Miyake (2004) further distinguished resistance to distractor interference from inhibition of prepotent responses. The former is commonly measured by interference costs in the Stroop task (MacLeod, 1991), whereas the latter is commonly examined using Go/NoGo or stop-signal paradigms (Bari and Robbins, 2013; Verbruggen et al., 2019).

The distinction between interference control and response inhibition is especially relevant for badminton. In the Stroop task, the critical demand is to select the task-relevant stimulus dimension while resisting interference from a competing dimension. In badminton, a conceptually similar demand arises when athletes prioritize diagnostic cues and suppress misleading or low-value information from an opponent's feint, body motion, or deceptive racket action. In the Go/NoGo task, the critical demand is to withhold or cancel a prepared response when a NoGo signal appears. A conceptually related sport demand arises when a player prepares to move or strike but must inhibit that action after a late change in the opponent's shot or shuttle trajectory. Recent open-skill sport evidence also suggests that interference control and response inhibition can show partly different behavioral and neurophysiological profiles across sport contexts (Ludyga et al., 2022; Vona et al., 2024). Therefore, the present study treats the two tasks as complementary but non-equivalent probes of inhibitory-control performance.

Existing research on sport experts and open-skill sports has provided evidence for associations between sport experience and executive-function performance, but the conclusions are heterogeneous. Early work found that open-skill athletes often outperformed closed-skill athletes and non-athletes on inhibitory-control tasks (Wang et al., 2013). More recent systematic reviews, meta-analyses, and latent-variable studies show that athletes can perform better than control groups across cognitive tasks, but effects vary by task type, sport type, competitive level, sample characteristics, and index selection (Scharfen and Memmert, 2019; Brimmell et al., 2025; Simonet et al., 2023; Albaladejo-García et al., 2023; Furley et al., 2025; Ren et al., 2025). This mixed literature prevents a simple claim that open-skill sport training produces a general executive-function advantage. A more defensible question is which task indices are associated with badminton expertise and whether the pattern is similar across interference control and response inhibition.

With respect to badminton, existing studies provide relevant but incomplete evidence. Chen et al. (2019) used stop-signal and change-signal tasks and reported more efficient behavioral and event-related potential characteristics during action inhibition and re-engagement processing in collegiate badminton athletes. Takahashi and Grove (2019) used a within-subjects design and found that acute badminton exercise facilitated Stroop performance. Chang et al. (2024) reported competitive-level differences in visuospatial attention, and Hu (2026) reported cross-sectional Flanker-task differences between adolescent badminton trainees and non-athletes. These studies suggest that badminton expertise is relevant to cognitive-control performance, but they differ in task domain, time scale, age group, and outcome measure. The present study does not introduce a new inhibitory-control paradigm or estimate latent inhibitory-control ability. Rather, it applies two standard laboratory tasks to the same expert-novice sample to examine whether task-based group differences appear for both stimulus-level interference control and action-level response inhibition.

Based on this evidence, the present study examined whether badminton expertise was associated with performance differences on two non-sport-specific cognitive-control tasks. Experiment 1 used the participant-level Stroop effect as its principal index. Experiment 2 triangulated raw Go hit and NoGo false-alarm rates with a trial-level NoGo-error model; corrected d′ was retained as a secondary composite because ceiling hit rates and floor false-alarm rates constrain its information. Go reaction time, condition-level Stroop accuracy and reaction time, inverse-efficiency scores, and standardized cross-task scores were secondary or exploratory. All predictions concern cross-sectional associations with expertise rather than causal effects of training.

## 2 Experiment 1: interference control

### 2.1 Participants

The original *a priori* calculation was performed in G^*^Power 3.1 for the planned 2 (group) × 2 (trial type) mixed-design ANOVA (α = 0.05, 1—β = 0.95, f = 0.25), which indicated a minimum total sample of 54 participants (Faul et al., 2007, 2009). This design-stage calculation was not tailored to the Welch *t* test of participant-level Stroop effects that was subsequently designated as the primary contrast in the rebuilt analysis. It is therefore reported as the original sampling justification rather than as a direct power analysis for the Welch test. The final sample comprised 68 participants: 33 collegiate badminton athletes with national second-class athlete status or above (*M* age = 20.7 years, SD = 1.3; M training history = 11.2 years, SD = 2.6; at least 15 h/week of training) and 35 students without specialized badminton training (M age = 20.8 years, SD = 1.6). The expert group comprised 16 male and 17 female participants; the novice group comprised 17 male and 18 female participants. All participants were right-handed, had normal or corrected-to-normal vision, had no color-vision deficiency, and reported no neurological disease. Written informed consent was obtained, participants were compensated, and the protocol was approved by the Biomedical Research Ethics Committee of Hunan Normal University (Approval No. 20251021) in accordance with the Declaration of Helsinki.

### 2.2 Materials

A classic color-word Stroop paradigm was used. The stimuli were the Chinese characters for “red,” “green,” and “yellow,” presented in red, green, or yellow, yielding 180 congruent and 180 incongruent formal trials in random order. The experiment was programmed and administered using PsychoPy version 2025.2.3. Keyboard responses were collected using the PsychoPy Builder Keyboard Component and a wired USB keyboard. Visual stimuli were presented on a 23.8-inch LCD monitor with a resolution of 1,920 × 1,080 pixels and a refresh rate of 60 Hz. All participants were tested using the same computer, keyboard, monitor, room, and experimental settings. The manufacturer and model of the keyboard were not recorded. No independent hardware-based validation of stimulus-presentation or response-recording timing was conducted. Therefore, although reaction times were recorded by PsychoPy and exported with millisecond numerical resolution, the setup was not independently verified to provide millisecond-level timing accuracy.

### 2.3 Procedure

**Figure 1:**
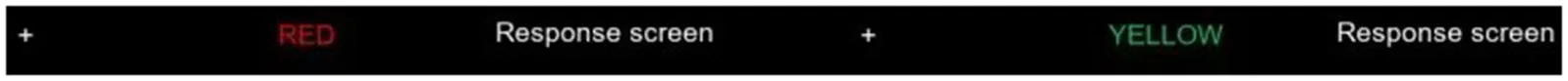
Stimulus sequence of the Stroop task.

Stroop task (see Figure 1): After reading the instructions, participants entered a practice phase (12 trials), followed by the formal testing phase (360 trials). Each trial proceeded as follows: a fixation cross “+” was presented at the center of the screen for 500 ms; a color-word stimulus was then presented for 1,000 ms, and participants were instructed to ignore the word meaning and judge the color as quickly as possible by pressing a key (red = “1,” green = “2,” yellow = “3”); after the stimulus disappeared, a blank response screen was presented for 2,000 ms, during which keypresses were valid. The intertrial interval was 1,000 ms. No feedback was provided, and participants were instructed to respond as quickly and accurately as possible. The Stroop task was administered before the Go/NoGo task for all participants; therefore, task order was fixed rather than counterbalanced.

### 2.4 Data collection and analysis

PsychoPy recorded trial-level reaction time (RT) and accuracy. Accuracy was the percentage of correct responses among all trials. RT analyses used correct responses with a recorded RT. For the cleaned analysis, RTs below 100 ms or beyond each participant-by-condition mean ± 3 SD were excluded. This removed 387/24,480 trials (1.58% of all trials; 1.64% of correct trials with a recorded RT): 172/11,880 expert trials (1.45%) and 215/12,600 novice trials (1.71%). The participant-level Stroop effect was mean incongruent RT minus mean congruent RT. A sensitivity analysis recomputed the effect from all correct trials with a recorded RT, without the RT threshold or ± 3-SD trimming. Supplementary Figure 1 displays the within-person trial spread for both conditions and the participant-level effects.

The original mixed-design analyses were conducted in SPSS 26.0. Revised analyses used Python 3.12.7 (pandas 2.2.2, SciPy 1.13.1, statsmodels 0.14.2) and R 4.6.0 with lme4 2.0–1. For Experiment 1, 2 (group) × 2 (trial type) mixed-design ANOVAs were conducted for accuracy and cleaned RT. The participant-level Stroop effect was compared with Welch's *t* test because variances differed; Mann-Whitney U and untrimmed analyses assessed robustness. Ten thousand participant-level bootstrap resamples within group produced percentile 95% CIs for means, contrasts, and Cohen's d. Speed sensitivity was assessed with an ANCOVA of the cleaned Stroop effect on group and cleaned Go RT using HC3 standard errors; a group × Go RT term tested slope homogeneity. An inverse-efficiency Stroop effect, RT/accuracy for incongruent minus congruent trials, was also analyzed. Split-half reliability was estimated using odd-even trials and 1,000 random condition-stratified halves; Pearson correlations were corrected with the Spearman-Brown formula and reported pooled, within group, and after group-mean adjustment. No manuscript-wide multiplicity correction was applied across distinct outcomes; Bonferroni adjustment was limited to the planned simple-effects comparisons, and secondary/exploratory *p* values are interpreted cautiously.

### 2.5 Results

Descriptive statistics for accuracy are shown in Table 1. Accuracy was high in both groups; experts averaged 96.55% on congruent and 96.01% on incongruent trials, whereas novices averaged 96.89% and 95.29%, respectively.

**Table 1:** Accuracy of the two groups on congruent and incongruent trials (%, M ± SD).

Group	*n*	Congruent trials	Incongruent trials
Expert	33	96.55 ± 2.72	96.01 ± 3.07
Novice	35	96.89 ± 2.38	95.29 ± 3.11

The accuracy ANOVA (Table 2) showed no group main effect, *F*_(1, 66)_ = 0.09, *p* = 0.760, ηp^2^ < 0.01. Accuracy was lower on incongruent than congruent trials, *F*_(1, 66)_ = 13.97, *p* < 0.001, ηp^2^ = 0.17. The group × trial-type interaction did not reach α = 0.05, *F*_(1, 66)_ = 3.45, *p* = 0.068, ηp^2^ = 0.05.

**Table 2:** Mixed-design ANOVA results for accuracy.

Source	df1,df_2_	*F*	*p*	ηp^2^
Group	1, 66	0.09	0.760	< 0.01
Trial type	1, 66	13.97	< 0.001	0.17
Group × trial type	1, 66	3.45	0.068	0.05

Cleaned correct-trial RTs are shown in Table 3 and reported to the nearest millisecond. Experts averaged 570 ms on congruent and 584 ms on incongruent trials; novices averaged 591 ms and 636 ms, respectively.

**Table 3:** Clean correct-trial reaction times by group and trial type (ms, M ± SD).

Group	*n*	Congruent trials	Incongruent trials
Expert	33	570 ± 69	584 ± 66
Novice	35	591 ± 54	636 ± 59

**Figure 2:**
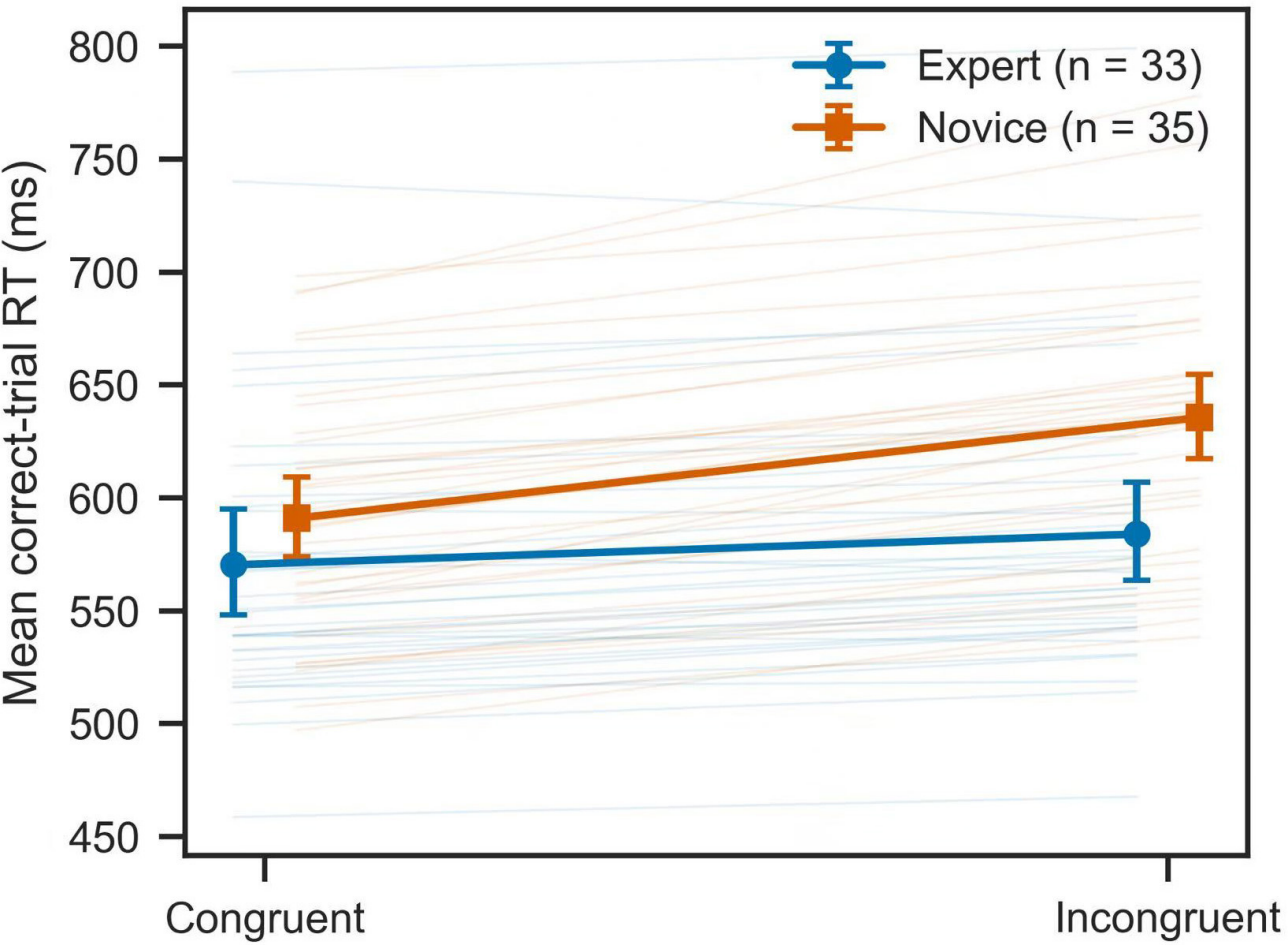
Interaction plot for reaction time.

The RT ANOVA (Table 4) showed a group main effect, *F*_(1, 66)_ = 5.85, *p* = 0.018, ηp^2^ = 0.08, a trial-type main effect, *F*_(1, 66)_ = 325.81, *p* < 0.001, ηp^2^ = 0.83, and a group × trial-type interaction, *F*_(1, 66)_ = 92.38, *p* < 0.001, ηp^2^ = 0.58. Thus, the expert-novice difference was larger on incongruent trials than on congruent trials (Figure 2).

**Table 4:** Mixed-design ANOVA results for reaction time.

Source	df_1_,df_2_	*F*	*p*	ηp^2^
Group	1, 66	5.85	0.018	0.08
Trial type	1, 66	325.81	< 0.001	0.83
Group × trial type	1, 66	92.38	< 0.001	0.58

Bonferroni-adjusted simple effects showed longer incongruent than congruent RTs within experts (Figure 2), *t* (32) = 7.65, *p* < 0.001, mean difference = 14 ms, and within novices, *t* (34) = 16.87, *p* < 0.001, mean difference = 45 ms. The groups did not differ significantly on congruent RT, Welch *t* (60.80) = 1.37, *p* = 0.174, mean difference = 21 ms, but novices were slower on incongruent RT, Welch *t* (63.96) = 3.40, *p* = 0.001, mean difference = 52 ms. Because response-device timing was not externally validated, these software-timestamp differences should not be interpreted as hardware-validated millisecond accuracy.

**Figure 3:**
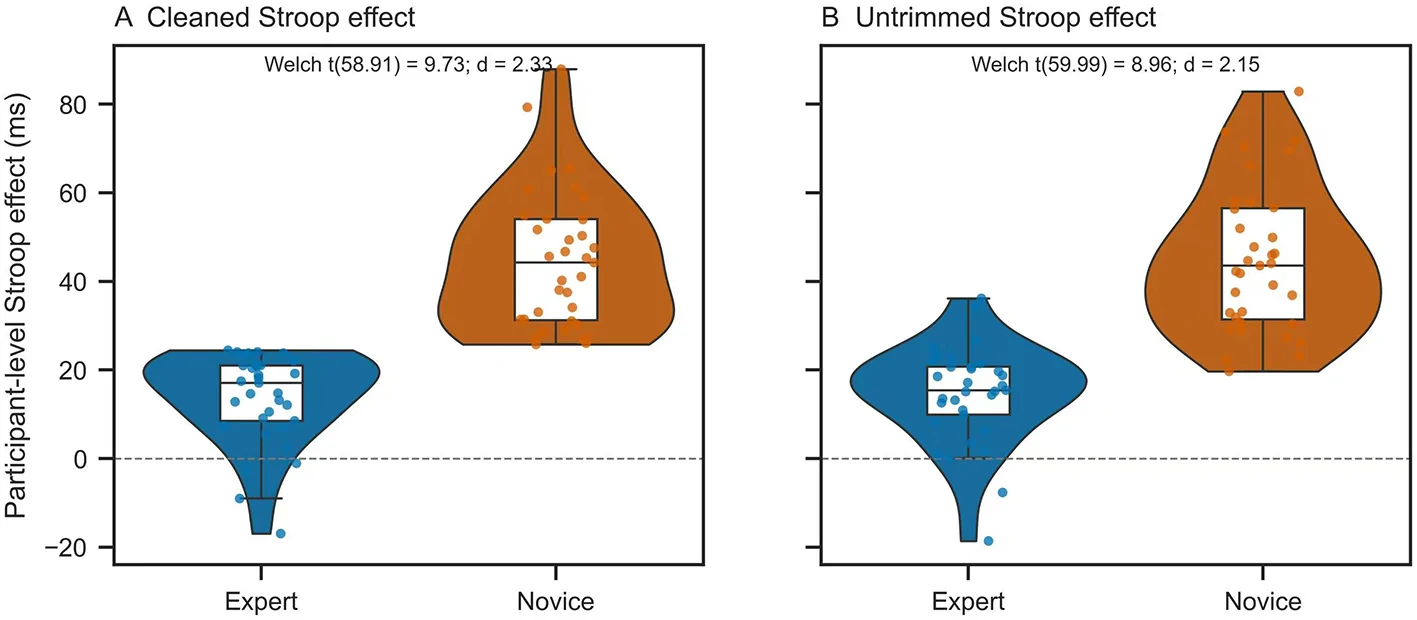
Rebuilt participant-level distributions of the cleaned **(A)** and untrimmed **(B)** Stroop effects. Each point represents one participant; violins show the distribution and boxes show the median and interquartile range. Both panels were generated from the authoritative Stroop trial-level file. The cleaned expert distribution includes negative values (range,−16.9 to 24.4 ms).

For the cleaned Stroop effect (Table 5; Figure 3A), experts averaged 13.6 ms [SD = 10.2, 95% bootstrap CI (10.0, 16.8)] and novices averaged 44.6 ms [SD = 15.6, 95% CI (39.8, 49.9)]. The novice-minus-expert difference was 31.0 ms [95% CI (25.2, 37.3)], Welch *t* (58.91) = 9.73, *p* < 0.001, Cohen's *d* = 2.33 [95% CI (1.99, 2.88)]. Cleaned full-length effects did not overlap (expert range = −16.9 to 24.4 ms; novice range = 25.8 to 87.9 ms). However, without any RT trimming (Figure 3B), the groups did overlap (expert range = −18.6 to 36.1 ms; novice range = 19.7 to 82.8 ms) and the difference remained significant: 30.4 ms [95% CI (24.0, 37.0)], Welch *t* (59.99) = 8.96, *p* < 0.001, *d* = 2.15 [95% CI (1.77, 2.69)].

**Table 5:** Cleaned participant-level Stroop effects by group (ms, M ± SD).

Group	*n*	Mean	SD	*t*	df	Cohen's *d*
Expert	33	13.6	10.2	9.73	58.91	2.33
Novice	35	44.6	15.6			

Positive *t* and Cohen's *d* values are reported as absolute effect sizes; the direction of group differences is described in the text.

The cleaned effect departed from normality in experts, *W* = 0.880, *p* = 0.002, and novices, *W* = 0.923, *p* = 0.017; Brown-Forsythe's test also indicated unequal variances, *F*_(1, 66)_ = 5.73, *p* = 0.020. The Mann-Whitney test confirmed the group difference, *U* = 1,155, *p* < 0.001. In the speed-control ANCOVA, the adjusted novice-minus-expert contrast remained 29.1 ms [HC3 95% CI (22.8, 35.5), *p* < 0.001]; Go RT was not a significant covariate (*p* = 0.222), and the group × Go RT slope test was non-significant (*p* = 0.188). The inverse-efficiency contrast also favored experts [mean difference = 39.3 ms, 95% CI (29.6, 49.8), *d* = 1.80]. Split-half reliability estimates for the Stroop effect and corrected d′ are summarized in Supplementary Table 1.

### 2.6 Discussion

Experiment 1 used a Stroop color-word task to examine whether collegiate badminton athletes differed from novices in a laboratory index of interference control. The behavioral results showed no significant difference in overall accuracy between the expert and novice groups. In reaction time, the expert group responded faster than the novice group, and this difference was concentrated on incongruent trials. The smaller Stroop effect in the expert group is consistent with lower conflict-related slowing. However, this task-based result should not be interpreted as direct evidence for a general inhibitory-control ability, because the Stroop effect can also reflect processing speed, response mapping, alertness, task strategy, and speed-accuracy tradeoffs.

The clean and untrimmed analyses both support a sample-specific group difference in conflict-related slowing, and the difference remained after controlling for Go RT and in inverse-efficiency scores. The full-length cleaned distributions were separated because each effect averaged approximately 340 retained correct trials and the between-group mean shift was large relative to the final standard errors. This separation does not imply reliable individual ranking: group-adjusted and within-group split-half correlations were low, and the untrimmed distributions overlapped. The result may still reflect self-selection, processing or motor speed, response mapping, fitness, alertness, motivation, keyboard familiarity, or other unmeasured characteristics and should not be generalized to a unitary inhibitory-control ability.

## 3 Experiment 2: response inhibition

### 3.1 Participants

Same as Experiment 1.

### 3.2 Materials

A standard Go/NoGo paradigm was used. The stimuli were a white triangle (Go stimulus) and a blue triangle (NoGo stimulus). The formal experiment contained 320 trials, including 240 Go trials (75%) and 80 NoGo trials (25%), presented in random order. The experimental program, monitor, response device, testing room, and computer setup were the same as those used in the Stroop task.

### 3.3 Procedure

**Figure 4:**
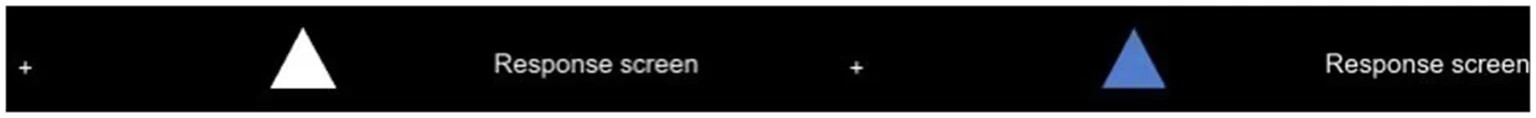
Stimulus sequence of the Go/NoGo task.

Go/NoGo task (Figure 4): After reading the instructions, participants entered a practice phase (10 trials), followed by the formal testing phase (320 trials). Each trial proceeded as follows: a fixation cross “+” was presented at the center of the screen for 500 ms; a white or blue triangle was then presented for 1,000 ms, and participants were instructed to press the space bar quickly in response to the white triangle and withhold responses to the blue triangle; after the stimulus disappeared, a blank response screen was presented for 2,000 ms, during which key presses were valid. The intertrial interval was 1,000 ms. No feedback was provided. This task was always completed after the Stroop task, so the Go/NoGo results may be more vulnerable to task-order, endurance, and fatigue effects.

### 3.4 Data collection and analysis

**Figure 5:**
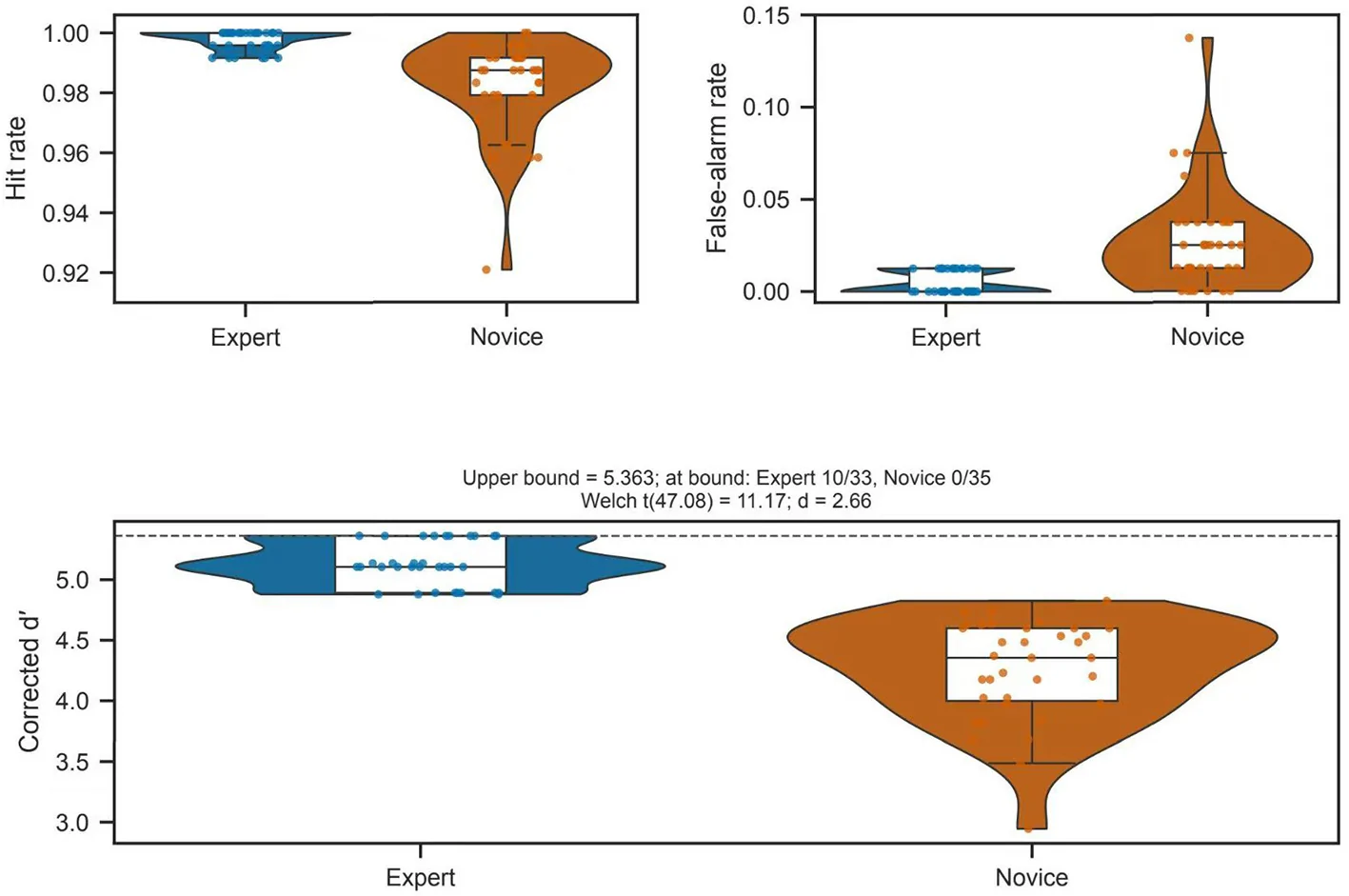
Rebuilt participant-level distributions of hit rate, false-alarm rate, and corrected d′ from the authoritative Go/NoGo trial-level file. Each point represents one participant; violins show the distribution and boxes show the median and interquartile range. The dashed line marks the correction-imposed upper d′ bound of 5.363; 10/33 experts and 0/35 novices attained the bound.

For each participant, hit rate was the proportion of correct Go responses and false-alarm rate was the proportion of erroneous NoGo keypresses. Correct Go RTs were summarized after the archived Go-RT cleaning rule. Corrected d′ was computed as *z* (hit rate)—*z* (false-alarm rate), using the 1/(2N) correction for proportions of 0 or 1 within each response category. With 240 Go and 80 NoGo trials, this rule imposes an upper bound of d′ = 5.363 when the observed hit rate is 1 and the false-alarm rate is 0. Because the observed hit rates approached ceiling and false-alarm rates approached floor, d′ is treated as a secondary, upper-censored composite and interpreted jointly with the raw distributions. Figure 5 displays participant-level hit, false-alarm, and corrected-d′ distributions.

Go RT, hit rate, false-alarm rate, and corrected d′ were compared with Welch tests, Mann-Whitney sensitivity tests, and 10,000 participant-level bootstrap resamples. The principal trial-level analysis modeled each NoGo error with a binomial-logit mixed-effects model including group as a fixed effect and a participant random intercept, fitted in lme4 with the bobyqa optimizer. This model preserves the trial as the observation while accounting for repeated trials within participants. Split-half d′ was computed by dividing Go and NoGo trials separately, applying the same extreme-rate correction within each half, correlating half scores, and applying the Spearman-Brown correction.

### 3.5 Results

Table 6 and Figure 5 report the Go RT, hit-rate, false-alarm-rate, and corrected-d′ results. The participant-level distributions underlying these results are additionally presented in Supplementary Figure 2. The participant distributions reveal marked ceiling hit rates and floor false-alarm rates in experts.

**Table 6:** Go/NoGo participant-level indices by group (M ± SD).

Measure	Expert, M ±SD	Novice, M ±SD	Direction-labeled contrast [95% bootstrap CI]	Welch *t* (df), *p*	Cohen's *d* [95% CI]
Go RT (ms)	259 ± 38	290 ± 58	Novice– expert = 30.8 [8.2, 54.7]	2.60 (58.35), 0.012	0.62 [0.18, 1.10]
Hit rate	0.9976 ± 0.0033	0.9825 ± 0.0160	Expert –Novice = 0.0151 [.0102,0.0209]	5.46 (37.05), < 0.001	1.29 [1.02, 1.79]
False-alarm rate	0.0049 ± 0.0062	0.0264 ± 0.0276	Novice– Expert =0.0215 [.0130,0.0315]	4.49 (37.63), < 0.001	1.06 [0.79, 1.57]
Corrected d′	5.13 ± 0.18	4.26 ± 0.42	Expert – Novice = 0.87 [0.73, 1.02]	11.17 (47.08), < 0.001	2.66 [2.24, 3.36]

Experts had faster cleaned Go RTs (M = 259 ms, SD = 38) than novices (M = 290 ms, SD = 58), mean difference = 30.8 ms [95% bootstrap CI (8.2, 54.7)], Welch t (58.35) = 2.60, *p* = 0.012, *d* = 0.62 [95% CI (0.18, 1.10)]. Hit rates were.9976 [SD = 0.0033, median = 1.000, IQR (0.9958, 1.000), range (0.9917, 1.000)] in experts and 0.9825 [SD = 0.0160, median = 0.9875, IQR (0.9792, 0.9917), range (0.9208, 1.000)] in novices; the expert-minus-novice difference was 0.0151 [95% CI (0.0102, 0.0209)], *p* < 0.001, *d* = 1.29. False-alarm rates were 0.0049 [S = 0.0062, median = 0.0000, IQR (0.0000, 0.0125), range [0.0000, 0.0125)] in experts and 0.0264 [SD = 0.0276, median = 0.0250, IQR (0.0125, 0.0375), range (0.0000, 0.1375)] in novices; the novice-minus-expert difference was 0.0215 [95% CI (.0130,0.0315)], Welch *t* (37.63) = 4.49, *p* < 0.001, *d* = 1.06. Corrected d′ was 5.13 [SD = 0.18, 95% CI (5.07, 5.19)] in experts and 4.26 [SD = 0.42, 95% CI (4.11, 4.39)] in novices; the difference was 0.87 [95% CI (0.73, 1.02)], Welch *t* (47.08) = 11.17, *p* < 0.001, *d* = 2.66 [95% CI (2.24, 3.36)]. The correction-imposed upper bound was 5.363; 10/33 experts and 0/35 novices attained it.

The Go/NoGo participant-level indices were non-normal (all Shapiro-Wilk *p* ≤ 0.008), and Mann-Whitney sensitivity tests gave the same directional conclusions. The trial-level mixed model included 5,440 NoGo trials and converged without a singular fit. Experts made 13/2,640 errors and novices 74/2,800 errors; the expert coefficient was β = −1.68 (SE = 0.34), *z* = −4.91, *p* < 0.001, corresponding to OR = 0.19 [95% CI (0.10, 0.36)]; participant random-intercept SD = 0.61. Odd-even corrected d′ reliability was 0.81 pooled [95% CI (.66,0.90)] and 0.58 after group adjustment [95% CI (.07,0.79)], but the expert-only raw half correlation was negative (*r* = −0.14; corrected *r* = −0.33), consistent with ceiling/floor range restriction. The novice-only corrected estimate was 0.65. Across random stratified splits, median corrected reliability was 0.74 pooled, 0.40 group-adjusted,−0.72 in experts, and 0.49 in novices.

Contrasts are direction-labeled; Cohen's d values are absolute magnitudes. Percentile bootstrap CIs are based on 10,000 participant-level resamples.

### 3.6 Discussion

Experiment 2 showed faster Go responses, higher hit rates, and fewer NoGo errors in experts. The trial-level mixed model confirmed that the false-alarm contrast was not an artifact of reducing trials to a single participant score. Nevertheless, hit rates were at ceiling and false-alarm rates were at floor, especially among experts. Under these conditions, corrected d′ mainly separates “at ceiling” from “near ceiling,” compresses within-group variance, and is weakly informative about individual discriminability. It is therefore retained only as a secondary composite and must be interpreted alongside raw hit/false-alarm distributions and the trial-level model.

Unlike the interference control measured by the Stroop task, the Go/NoGo task places greater emphasis on action-level response inhibition (Bari and Robbins, 2013; Verbruggen et al., 2019). Rapid jumping, abrupt stopping, directional changes, and responses to feints in badminton may require athletes to decide whether to execute or cancel an action under time pressure. The present findings are consistent with the possibility that badminton expertise is associated with more accurate response discrimination and fewer erroneous NoGo responses. However, because the expert group also responded faster on Go trials, the results cannot fully separate inhibition-specific performance from general processing speed, motor speed, alertness, or motivation.

### 3.7 Cross-experiment integrative analysis: comparing interference control and response inhibition

The de-identified trial-level Stroop and Go/NoGo files were available for this revision and were used to verify participant ID and group membership. The legacy Stroop summary file contained an inconsistent ID-group mapping. It was therefore excluded from cross-task pairing and from every rebuilt analysis. Every table and figure in this manuscript was generated from the two trial-level files, whose participant ID-group mappings agreed exactly. The validated participant-level cross-task scores are presented in Supplementary Figure 3. Cleaned Stroop effects were reverse-standardized and corrected d′ values were standardized so that higher scores indicated better task performance. A 2 (group) × 2 (index type) mixed-design ANOVA was retained as a descriptive comparison.

**Figure 6:**
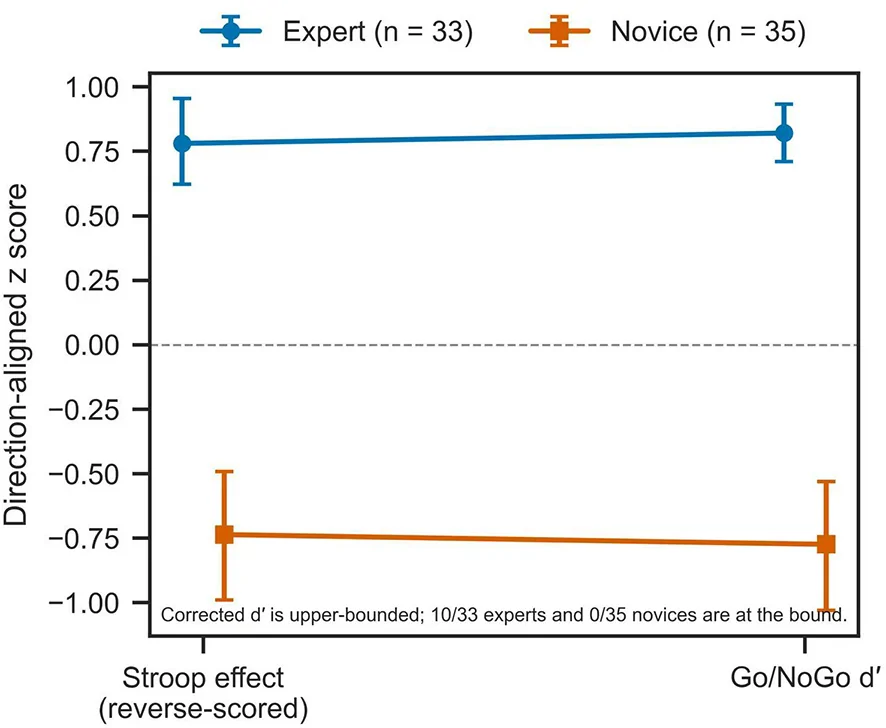
Descriptive interaction plot of direction-aligned standardized scores across the two task indices.

The revised cross-task analysis (Tables 7, 8) showed a group main effect, *F*_(1, 66)_ = 109.35, *p* < 0.001, ηp^2^ = 0.62, but no index-type main effect, *F*_(1, 66)_ < 0.01, *p* = 0.970, ηp^2^ < 0.01, and no group × index-type interaction, *F*_(1, 66)_ = 1.64, *p* = 0.204, ηp^2^ = 0.02 (Figure 6). The direction-aligned composite differed between groups (experts: *M* = 0.80, SD = 0.39; novices: *M* = −0.76, SD = 0.77), Welch *t* (51.18) = 10.64, *p* < 0.001, *d* = 2.54. Because corrected d′ is upper-censored and 10 experts sit at its bound, the standardized group effect (including ηp^2^ = 0.62) partly re-expresses the ceiling/floor censoring rather than providing independent evidence of a shared latent ability. A trial-level cross-task model was not fitted because the Stroop RT contrast and binary NoGo errors have non-equivalent outcome scales and error structures; the NoGo component is instead represented inferentially by the prespecified trial-level logistic mixed model. The standardized comparison is therefore descriptive only.

**Table 7:** Descriptive statistics for Z scores across inhibition types in the two groups (M ± SD).

Group	Index type	*n*	*M*	SD
Novice	Stroop effect (reverse)	35	−0.74	0.77
	Go/NoGo d′	35	−0.77	0.77
Expert	Stroop effect (reverse)	33	0.78	0.50
	Go/NoGo d′	33	0.82	0.34

**Table 8:** Mixed-design ANOVA results for group and inhibition type.

Source	df_1_,df_2_	*F*	*p*	ηp^2^
Group	1, 66	109.35	< 0.001	0.62
Index type	1, 66	< 0.01	0.970	< 0.01
Group × index type	1, 66	1.64	0.204	0.02

## 4 Discussion

### 4.1 Interference-control performance under high-conflict conditions

The present Stroop findings are best interpreted as a task-based interference-control association rather than as evidence for a global executive-function advantage (Brimmell et al., 2025; Furley et al., 2025; Manci et al., 2025). The expert group maintained high accuracy and showed its clearest reaction-time difference on incongruent trials, producing a smaller Stroop effect. Theoretically, this pattern is consistent with the idea that badminton expertise may be linked to faster selection of goal-relevant stimulus information when competing information is present. This interpretation fits the perceptual-cognitive demands of badminton, where players must extract useful opponent and shuttle cues while resisting misleading or irrelevant information (Chang et al., 2024; Hu, 2026). However, the absolute group difference in the Stroop effect was small and became a large standardized effect partly because the within-group SDs were small. Self-selection, general motor speed, physical conditioning, attentional endurance, response-device timing, RT preprocessing, and participant-level characteristics remain plausible contributors (Manci et al., 2025; Vona et al., 2024).

The absence of a significant group difference in accuracy remains informative. Overall accuracy was high in both congruent and incongruent conditions, suggesting a possible ceiling effect that may have compressed between-group differences. Under such conditions, reaction time and interference cost can reveal differences in the temporal cost of completing the task (Manci et al., 2025). The expert group's faster responses under comparable accuracy are therefore consistent with more efficient task performance. However, because no simple reaction-time baseline was included, this efficiency interpretation cannot be fully separated from group differences in general response speed (Vona et al., 2024).

### 4.2 Response-inhibition task performance: action cancellation and signal discrimination

In the Go/NoGo task, the expert group showed shorter Go reaction times, a lower NoGo false-alarm rate, and higher corrected discriminability. This pattern is consistent with better performance in a task that requires response discrimination and withholding of an inappropriate action (Albaladejo-García et al., 2023; Ludyga et al., 2022). In badminton terms, the result is compatible with the repeated need to prepare rapid actions while remaining able to stop or revise them after feints, deceptive strokes, or late trajectory changes (Chen et al., 2019; Hu, 2026). Nevertheless, corrected d′ was upper-censored: 10/33 experts and 0/35 novices attained the maximum value allowed by the 1/ (2N) correction. The faster Go reaction time means that response execution also differed between groups, and the low false-alarm rate suggests that the task approached floor performance for some participants. These data are therefore best described as a behavioral association between badminton expertise and Go/NoGo task performance, rather than as direct evidence that training strengthened action-inhibition mechanisms (Furley et al., 2025; Simonet et al., 2023).

### 4.3 Cross-task comparison: similarities, differences, and measurement constraints

The direction-aligned *Z*-score comparison showed group differences on both selected task indices, but the revised group × index interaction was not significant. Standardization does not make the Stroop effect and d′ equivalent in reliability, distribution, or psychological meaning. The low group-adjusted reliability of the Stroop effect and the correction-imposed upper censoring of d′ mean that the large pooled group effect, including ηp^2^ = 0.62, partly re-expresses group separation and censoring. The corresponding odd-even split-half score distributions are shown in Supplementary Figure 4. It cannot be interpreted as reliable individual ranking or a unitary latent-control construct. No manuscript-wide multiplicity correction was applied; Bonferroni adjustment was used only for the planned Stroop simple effects, and the cross-task analysis remains exploratory.

Taken together, the contribution of this study is not to demonstrate a unitary general inhibitory-control advantage. Rather, the study shows that the same group of collegiate badminton athletes performed better than novices on two standard, non-sport-specific tasks that are commonly used to index different inhibitory-control demands. The theoretical link is that badminton may place repeated demands on both stimulus-level filtering and action-level withholding, but the present data cannot determine whether these laboratory differences arise from training, self-selection, or correlated participant characteristics (Ludyga et al., 2022; Vona et al., 2024). This pattern is useful because it connects badminton expertise to two separable task indices within one sample while also showing why the interpretation must remain bounded.

## 5 Limitations and future directions

Several limitations constrain the interpretation of the present findings. First, the study used a cross-sectional expert-novice design. It therefore cannot determine whether badminton training improved inhibitory-control task performance or whether individuals with stronger baseline cognitive or motor characteristics were more likely to reach high competitive levels (Furley et al., 2025; Simonet et al., 2023). Longitudinal, intervention, or talent-selection designs are needed to separate training effects from self-selection.

Second, the study did not include a closed-skill athlete group or a physically active non-badminton group, so badminton-specific expertise cannot be separated from general athletic training, fitness, or competitive-sport participation. The sex distribution was 16 male and 17 female participants in the expert group and 17 male and 18 female participants in the novice group. However, BMI, sleep, fatigue, caffeine use, testing time, academic major, physical activity, and other sport experience were not systematically available and remain potential confounds.

Third, the design did not include a dedicated simple-RT or baseline processing-speed task. The revised Go-RT ANCOVA and inverse-efficiency analysis showed that the Stroop group contrast persisted, but Go RT was measured in a different task after the Stroop task and is not a pure baseline. Residual differences in processing speed, motor speed, keyboard familiarity, alertness, or motivation therefore remain plausible.

Fourth, the experiment used PsychoPy version 2025.2.3 and the PsychoPy Builder Keyboard Component with a wired USB keyboard, but the keyboard manufacturer and model were not recorded. No independent hardware-based validation of stimulus-presentation or response-recording timing was conducted. A 60-Hz display constrains visual timing to approximately 16.7-ms frames, while USB keyboard and software layers can add latency and variability. Accordingly, RTs have millisecond numerical resolution in the PsychoPy export but should be interpreted as relative software-timestamp differences within a constant setup, not as independently verified millisecond-level timing accuracy.

Fifth, the study used decontextualized laboratory paradigms. The findings show associations between badminton expertise and performance on Stroop and Go/NoGo indices, but they do not establish equivalent advantages in badminton-specific decisions or real match performance (Furley et al., 2025; Manci et al., 2025).

Finally, no physiological or neural measures were collected. Interpretations about online monitoring, action cancellation, or neural inhibition should therefore be considered hypotheses for future EEG, neuroimaging, or sport-specific studies, not conclusions from the present behavioral data.

## 6 Conclusions

This cross-sectional study found that collegiate badminton experts differed from novices on selected Stroop and Go/NoGo performance measures. The Stroop contrast was robust to untrimmed, speed-covariate, and inverse-efficiency analyses, but within-group split-half reliability was low and the untrimmed distributions overlapped. Go/NoGo experts had higher hit rates and fewer NoGo errors, including in a trial-level mixed model, but ceiling/floor performance made corrected d′ weakly informative for individual discriminability. The evidence therefore supports task- and sample-specific associations with expertise, not causal effects of training or a general inhibitory-control ability.

## Data Availability

The original contributions presented in the study are included in the article/Supplementary material, further inquiries can be directed to the corresponding author/s.
