# Supplementary material for: Collegiate badminton expertise is associated with interference-control and response-inhibition task performance: a cross-sectional expert–novice study
Source: Front Psychol. 2026 Aug 20;17:1919654. doi: 10.3389/fpsyg.2026.1919654 (PMC13538109; doi:10.3389/fpsyg.2026.1919654)
Supplement: Supplementary file 1 [file Data_Sheet_1.pdf]

Supplementary Material

Collegiate Badminton Athletes Show Collegiate Badminton Expertise Is Associated With Interference-Control and Response-Inhibition Task Performance: A Cross-Sectional Expert–Novice Study

All values were recomputed from the de-identified trial-level Stroop and Go/NoGo files. RT = reaction time; IQR = interquartile range; SB = Spearman-Brown corrected correlation.

Supplementary Table S1. Split-half reliability of the two derived indices

Task	Split	Pooled SB r	Group-adjusted SB r	Expert SB r	Novice SB r
Stroop effect	Odd-even	.61	.05	-.54	.29
Stroop effect	Median of 1,000 random splits	.52	-.15	-.66	.07
Corrected d′	Odd-even	.81	.58	-.33	.65
Corrected d′	Median of 1,000 random splits	.74	.40	-.72	.49

Note. Negative corrected coefficients indicate that the corresponding within-group halves did not preserve participant rank order; they should not be interpreted as a meaningful negative trait reliability. Pooled estimates can be inflated by the stable expert-novice mean difference. Odd-even participant-bootstrap 95% CIs were [.31, .79] for the pooled Stroop estimate, [-.78, .50] for its group-adjusted estimate, [.66, .90] for pooled d′, and [.07, .79] for group-adjusted d′.

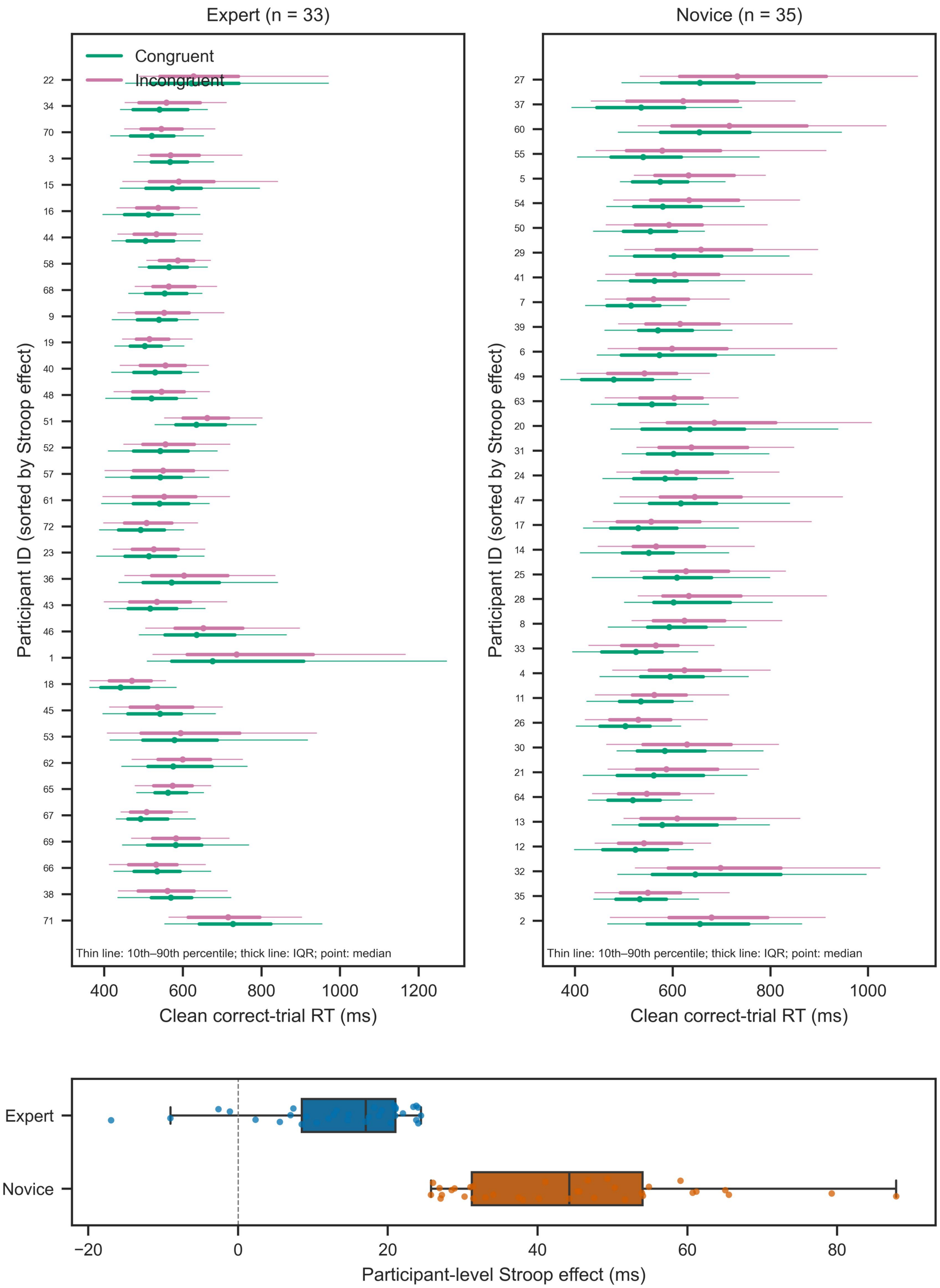

Supplementary Figure S1. Within-participant trial-score spread and participant-level Stroop effects. For each participant and condition, thin lines show the 10th-90th percentiles, thick lines the IQR, and points the median of retained correct-trial RTs. The lower panel shows full-length cleaned effects. Participants are sorted within group by the effect.

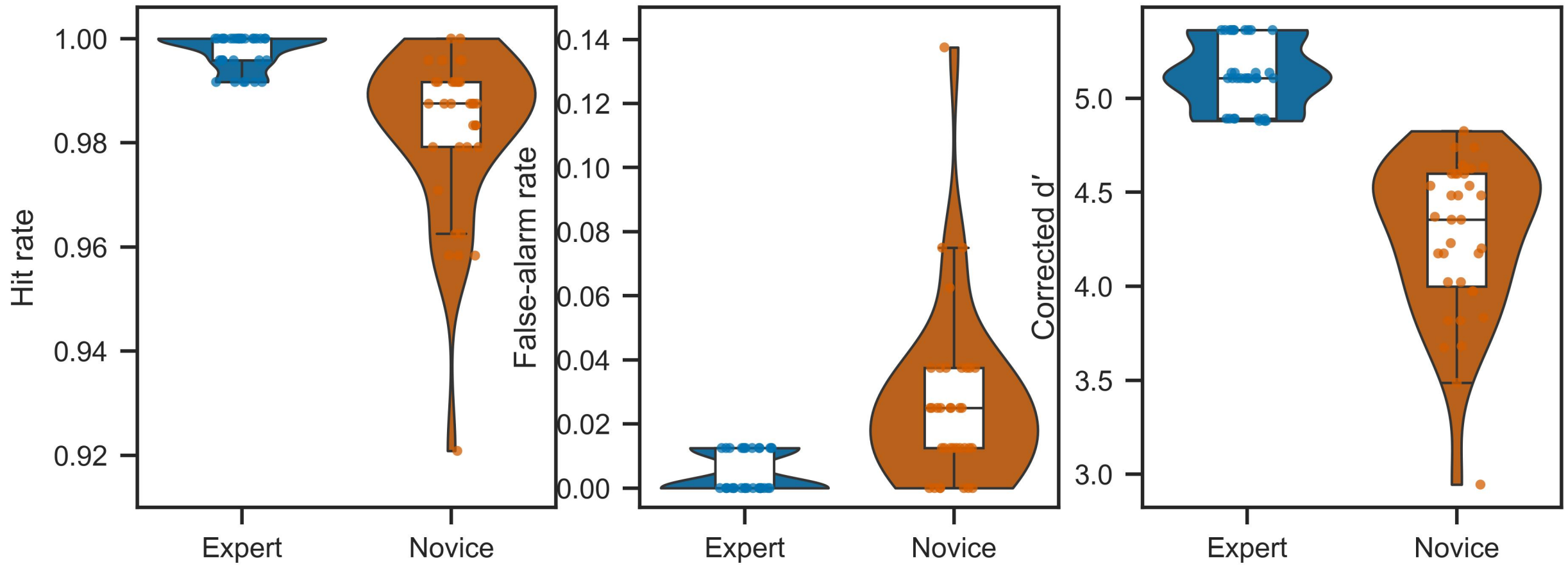

Supplementary Figure S2. Raw participant-level Go hit rates and NoGo false-alarm rates, with corrected d'. Points represent participants; violins and boxplots show the distributions. The ceiling/floor compression is visible in the expert group.

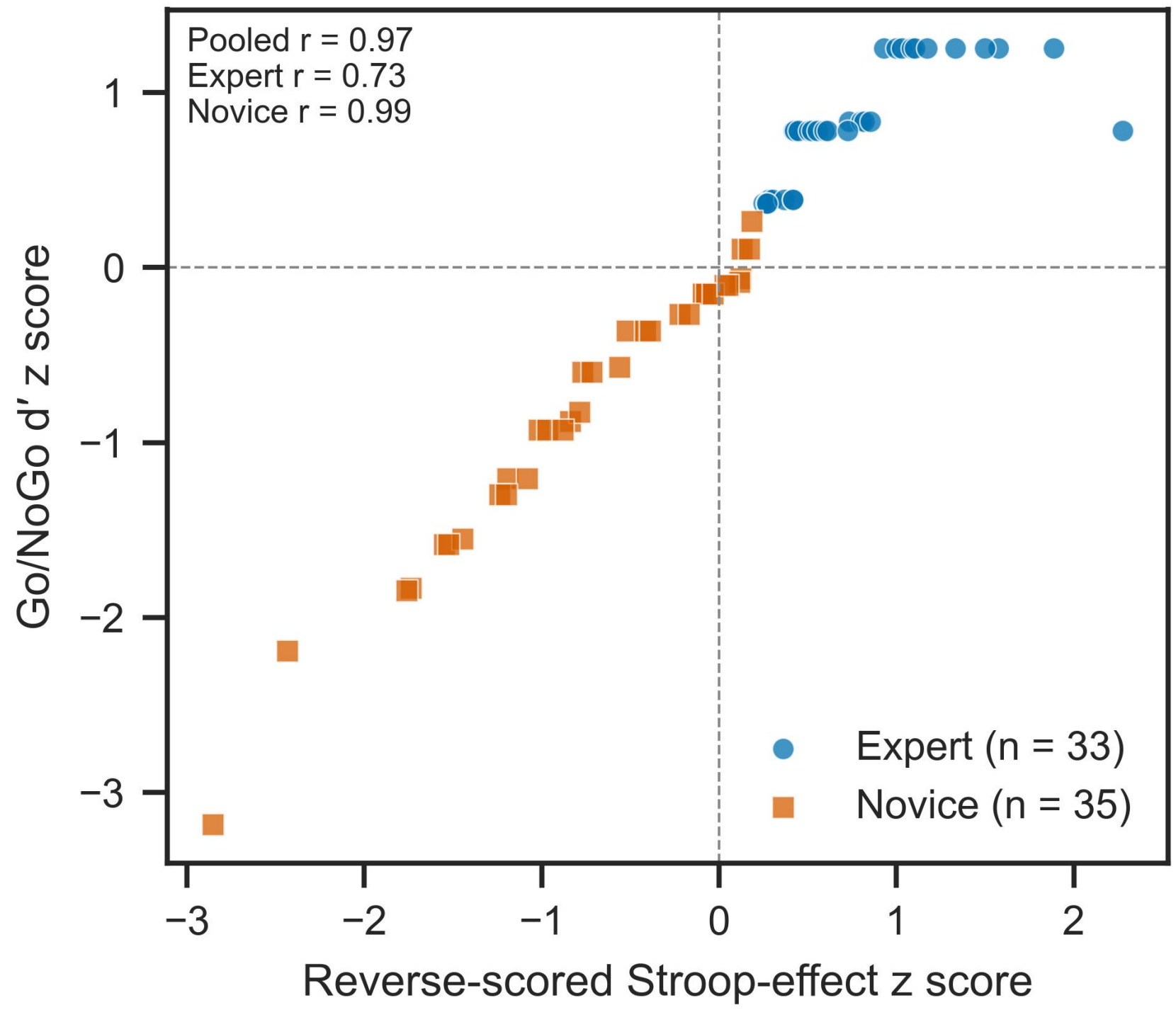

Supplementary Figure S3. Cross-task participant scores recomputed after validating participant ID and group mapping in the trial-level files. The Stroop effect was reverse-standardized; d' retained its original direction. Pooled correlations are dominated by group separation and should not be read as latent-trait validity.

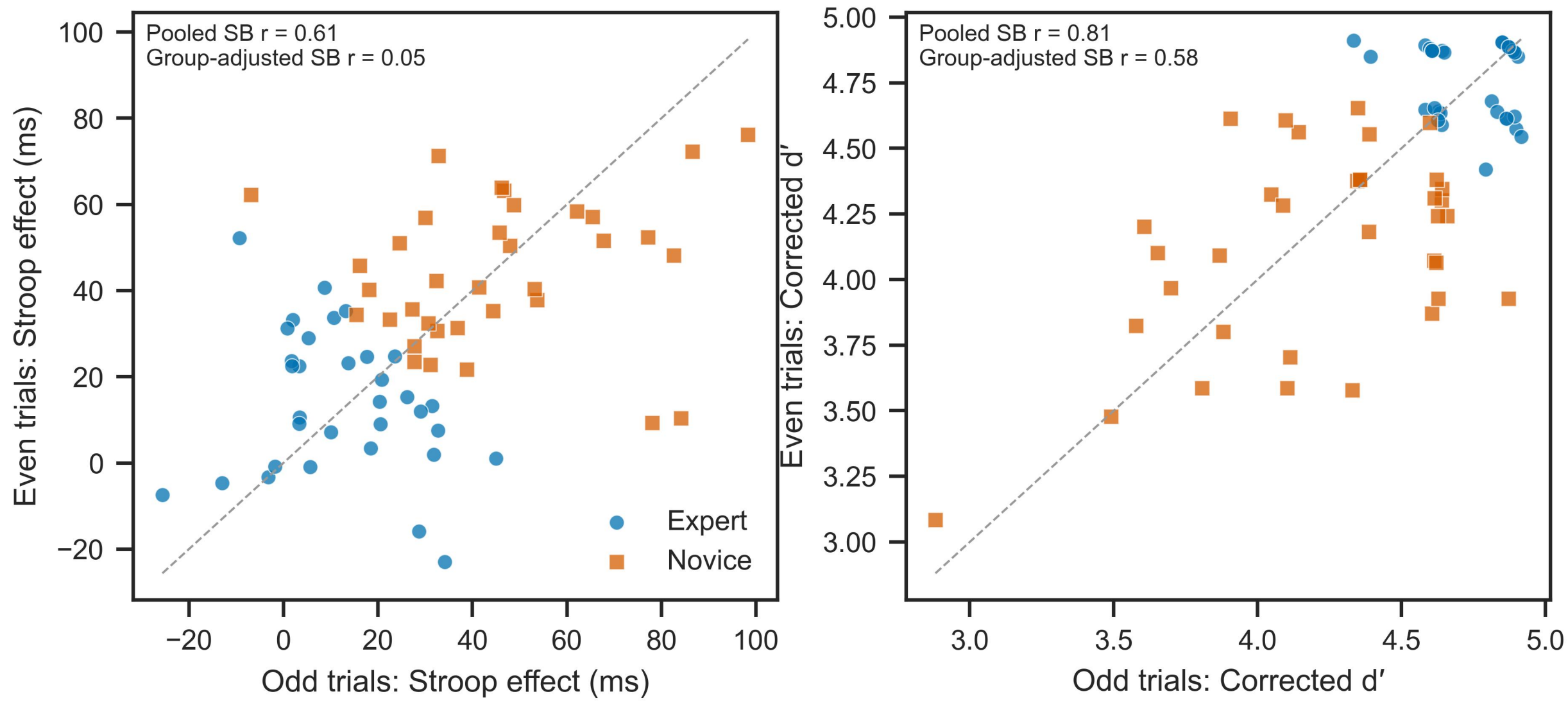

Supplementary Figure S4. Odd-even split-half scores. Pooled correlations partly reflect between-group separation; group-adjusted and within-group values quantify participant rank-order consistency after removing group means.

Data availability

The de-identified trial-level Stroop and Go/NoGo files, participant-level derived indices, analysis code, and analysis outputs are available from the corresponding author upon reasonable request.
